# OATP1B3 c.699G>A Predicts a 6.3-Fold Increased Risk of Hyperbilirubinemia During OPrD Therapy for HCV

**DOI:** 10.3390/cimb48050452

**Published:** 2026-04-27

**Authors:** Zuhal Altintas, Engin Altintas

**Affiliations:** 1 Department of Medical Genetics, Faculty of Medicine, Mersin University, 33343 Mersin, Turkey; 2 Department of Gastroenterology, Faculty of Medicine, Mersin University, 33343 Mersin, Turkey; enginaltintas@mersin.edu.tr

**Keywords:** antiviral agents, hepatitis C, chronic, direct-acting antivirals, hyperbilirubinemia, pharmacogenetics, solute carrier organic anion transporter family member 1B3

## Abstract

Although ombitasvir/paritaprevir/ritonavir plus dasabuvir (OPrD) therapy is highly effective for chronic hepatitis C (CHC), clinicians frequently encounter transient hyperbilirubinemia, which can be misidentified as hepatotoxicity. This study investigated the role of *SLCO1B1* (OATP1B1) and *SLCO1B3* (OATP1B3) genetic polymorphisms in predicting bilirubin spikes and distinguishing transporter-mediated interference from hepatocellular injury. In this prospective study of 65 patients with HCV genotype 1, genotyping for OATP1B1 (c.388A>G, c.521T>C) and OATP1B3 (c.334T>G, c.699G>A) was performed using PCR-RFLP and capillary electrophoresis (QIAxcel Advanced System). Clinical and biochemical parameters were monitored over a 12-week treatment period. Hyperbilirubinemia (total bilirubin >1.1 mg/dL) developed in 18.5% of the cohort, typically within the first month. A distinct ‘AST-dominant’ biochemical signature, elevated bilirubin and AST paired with stable ALT, was identified, suggesting transporter-specific interference rather than hepatocyte damage. Statistical analysis pinpointed the OATP1B3 c.699G>A (rs7311358) variant as the sole genetic driver (*p* = 0.007). Carriers of the c.699G>A allele faced a 6.3-fold higher risk of developing hyperbilirubinemia (OR: 6.30, 95% CI: 1.48–26.80, *p* = 0.032), while no significant associations were found for OATP1B1 variants. We conclude that OATP1B3 c.699G>A is a potent predictor of OPrD-induced hyperbilirubinemia. Identifying this genotype pre-treatment allows clinicians to anticipate transient, benign bilirubin elevations and prevent unnecessary drug discontinuation, thereby mitigating therapeutic inertia and ensuring treatment continuity for CHC patients.

## 1. Introduction

Bilirubin, the final product of heme degradation, circulates in an albumin-bound state until it is actively recruited across the sinusoidal membrane into hepatocytes for processing [1]. This critical hepatic uptake is primarily governed by the Organic Anion Transporting Polypeptide (OATP) family, specifically the OATP1B1 and OATP1B3 isoforms encoded by the *SLCO1B1* and *SLCO1B3* genes, respectively [2,3]. These solute carrier (SLC) transporters are indispensable for the sodium-independent uptake of endogenous compounds, such as unconjugated bilirubin and bile acids, as well as a diverse array of xenobiotics [4,5]. Consequently, any disruption in this transport machinery, whether triggered by cholestasis, competitive drug inhibition, or inherited genetic variants, compromises bilirubin clearance and leads to systemic accumulation [6,7,8].

The clinical management of chronic hepatitis C (CHC) has been revolutionized by direct-acting antiviral (DAA) regimens, most notably the combination of ombitasvir/paritaprevir/ritonavir and dasabuvir (OPrD) [9]. Despite achieving high virologic cure rates, the paritaprevir component serves as a potent inhibitor of both OATP1B1 and OATP1B3 [10]. This pharmacological blockade frequently induces transient, unconjugated hyperbilirubinemia in a subset of patients [10,11]. Although typically benign and self-limiting, these bilirubin spikes can be misidentified as signals of drug-induced liver injury (DILI), resulting in clinical concern or unwarranted treatment interruptions.

Emerging evidence suggests that the severity of this drug-induced biochemical shift is highly variable and likely anchored in the patient’s pharmacogenetic profile [12,13]. Single-nucleotide polymorphisms (SNPs) within the *SLCO* genes are known to reduce the expression or functional efficiency of OATP proteins, effectively lowering the threshold for competitive inhibition by DAAs [14]. While certain variants exhibit population-specific frequencies, their precise role in modulating the biochemical response to OPrD therapy remains insufficiently characterized in real-world clinical cohorts.

Given that OATPs dictate both bilirubin homeostasis and DAA kinetics, identifying genetic markers for treatment-related biochemical shifts is essential for personalized HCV management. This study evaluates the association between key *OATP1B1* (c.388A>G, c.521T>C) and *OATP1B3* (c.334T>G, c.699G>A) variants and the incidence of hyperbilirubinemia during OPrD therapy. By pinpointing these genetic drivers, we aim to establish a predictive framework that enhances patient safety, distinguishes transport interference from hepatocellular damage, and supports therapeutic continuity.

## 2. Materials and Methods

### 2.1. Study Population and Ethical Considerations

This prospective, single-center investigation was conducted at the Department of Gastroenterology, Mersin University Hospital (Mersin, Türkiye). The cohort comprised 65 patients diagnosed with Genotype 1 chronic hepatitis C (CHC). The study was conducted in strict accordance with the ethical principles of the 1964 Declaration of Helsinki and its subsequent amendments. Formal ethical approval was granted by the Clinical Research Ethics Committee of Mersin University (Approval No: 2016/194; Date: 9 June 2016). All participants provided written informed consent prior to enrollment and any study-related procedures.

Inclusion criteria were as follows: patients aged 18 to 90 years with confirmed chronic HCV Genotype 1 infection who were clinically eligible for the ombitasvir/paritaprevir/ritonavir plus dasabuvir (OPrD) treatment protocol. Exclusion criteria included refusal to participate, decompensated liver disease (Child-Pugh B or C), and HBV or HIV co-infections.

### 2.2. Treatment Regimen and Clinical Monitoring

Participants received a standard 12-week course of the OPrD regimen, consisting of a fixed-dose combination of ombitasvir (25 mg), paritaprevir (150 mg), and ritonavir (100 mg) once daily, supplemented by dasabuvir (250 mg) twice daily. For patients requiring ribavirin (RBV), weight-based dosing was initiated. RBV dosage was adjusted if hemoglobin levels decreased by ≥2 g/dL between baseline and Week 4, or if total hemoglobin concentration fell below 10 g/dL.

Clinical safety and therapeutic efficacy were rigorously monitored at baseline, Week 4, Week 12 (End of Treatment [EOT]), and Week 24 (Sustained Virological Response [SVR12]). Monitoring included quantitative HCV RNA assays (COBAS AmpliPrep/COBAS TaqMan HCV Test, v2.0), complete blood counts, and comprehensive biochemical profiling. Treatment-emergent hyperbilirubinemia was defined as any serum total bilirubin measurement exceeding 1.1 mg/dL during the 12-week treatment phase.

### 2.3. Genomic DNA Extraction and Genotyping Assays

Peripheral blood samples collected in EDTA tubes were stored at −20 °C until analysis. Genomic DNA was isolated using the PureLink™ Genomic DNA Kit (Thermo Fisher Scientific, Waltham, MA, USA) according to the manufacturer’s instructions. This study focused on four key functional polymorphisms: *SLCO1B1* (c.388A>G, rs2306283; c.521T>C, rs4149056) and *SLCO1B3* (c.334T>G, rs4149117; c.699G>A, rs7311358).

Genotyping was performed using the PCR-Restriction Fragment Length Polymorphism (PCR-RFLP) method. Specific primer sequences and restriction enzymes utilized for each variant are detailed in Supplementary Table S1 [15]. To ensure maximum precision and technical validation, fragment analysis was conducted using the QIAxcel Advanced System (QIAGEN, Hilden, Germany). This automated capillary electrophoresis platform provides superior resolution compared to traditional agarose gel electrophoresis. Genotype calling was standardized through the QIAxcel ScreenGel software (v1.6.0), which facilitated high-throughput accuracy and eliminated the risk of subjective visual interpretation bias.

### 2.4. Statistical Analysis

Statistical computations were executed using SPSS Statistics for Windows, Version 21.0 (IBM Corp., Armonk, NY, USA). Data normality was assessed using the Kolmogorov–Smirnov and Shapiro–Wilk tests. Normally distributed continuous variables are presented as mean ± standard deviation (SD), while non-parametric data are reported as medians with interquartile ranges (IQR). Categorical variables were analyzed using the Chi-square (χ^2^) test or Fisher’s exact test where appropriate.

Genotype frequencies were verified for Hardy–Weinberg Equilibrium (HWE). Inter-group comparisons were performed using Student’s *t*-test or the Mann–Whitney U test. To evaluate the genetic risk, multiple association models (Codominant, Dominant, and Recessive) were rigorously tested. The Akaike Information Criterion (AIC) and Bayesian Information Criterion (BIC) were calculated for each model to objectively determine the most parsimonious inheritance pattern. Based on these criteria and the relatively low frequency of the minor allele, a dominant inheritance model (wild-type vs. variant-allele carriers) was prioritized for the *SLCO1B3* c.699G>A locus to maximize statistical power and ensure a robust risk estimate. Odds Ratios (OR) with 95% Confidence Intervals (CI) were calculated to estimate the magnitude of risk. All analyses were two-tailed, and statistical significance was defined as *p* < 0.05.

Furthermore, a post hoc power analysis was conducted using G*Power software (v3.1.9.7) to validate the adequacy of the sample size (*n* = 65). Based on the 18.5% incidence of hyperbilirubinemia and the observed effect size (OR = 6.30) for the *OATP1B3* c.699G>A variant, the achieved statistical power was determined to be 0.81 (1-β). This exceeds the conventional threshold of 0.80, confirming that the study was sufficiently powered to detect the clinical impact of the investigated polymorphisms.

## 3. Results

### 3.1. Cohort Characteristics and Genotype Quality Control

This prospective study followed 65 patients (age range: 18–90 years) with Genotype 1 CHC throughout a 12-week OPrD treatment course. Compensated cirrhosis was present in 30.8% (*n* = 20) of the participants. During the observation period, treatment-emergent hyperbilirubinemia (total bilirubin > 1.1 mg/dL) manifested in 12 patients, representing an incidence of 18.5%.

**Figure 1:**
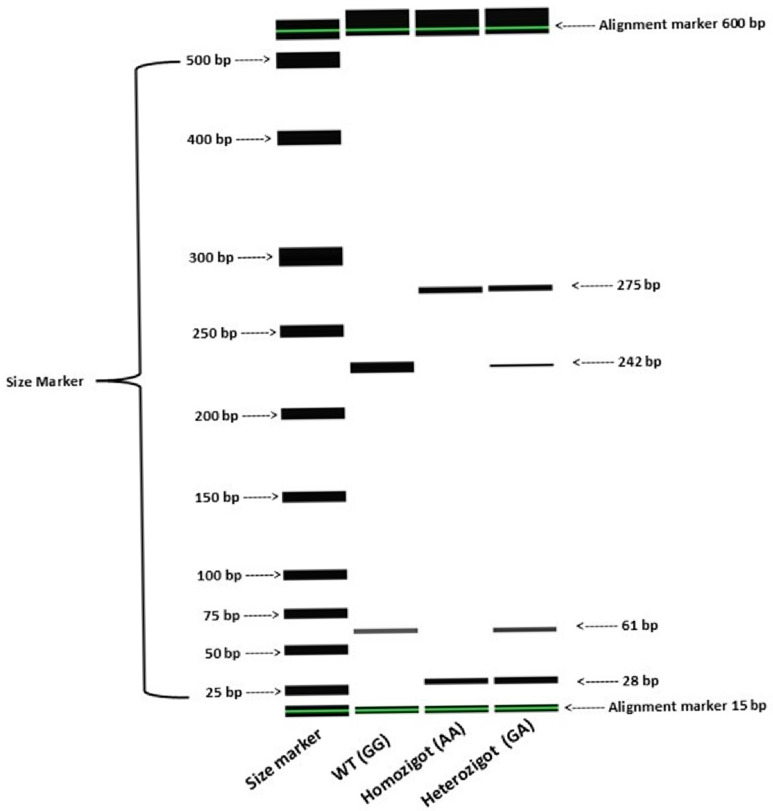

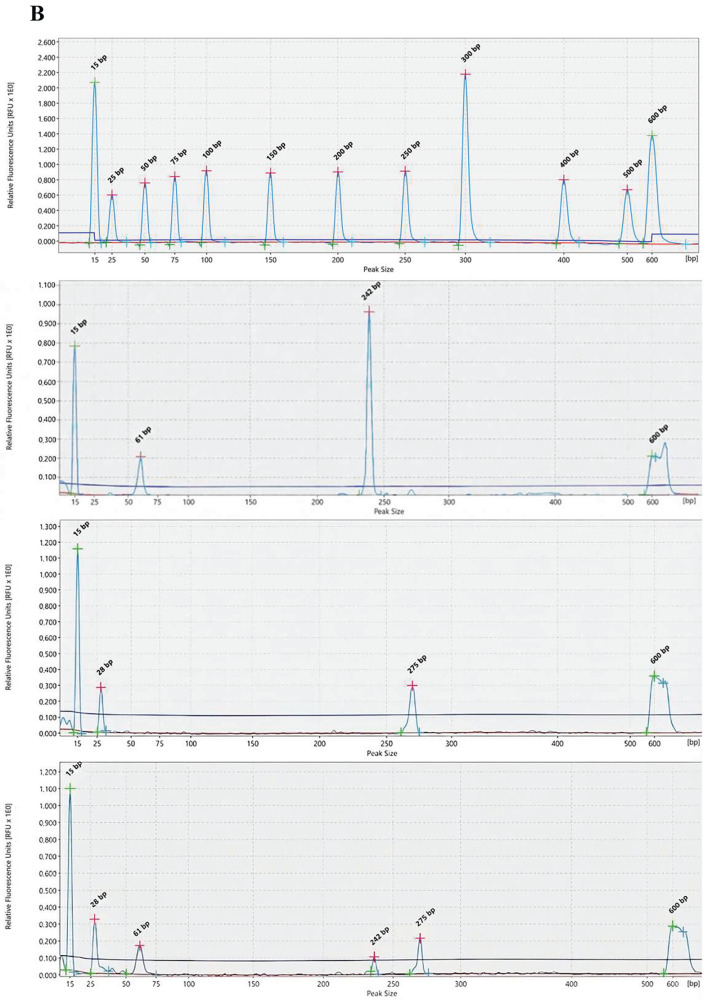
Technical validation of *SLCO1B3* c.699G>A (rs2110385) genotyping via automated capillary electrophoresis. (**a**) Digital gel image illustrating the distinct migration patterns for GG, GA, and AA genotypes. Green horizontal lines indicate internal alignment markers (15 bp and 600 bp), and black bands represent the target PCR products. (**b**) Electropherogram profiles providing a high-resolution view of the fluorescence intensity and peak distribution for each genotype. The vertical arrangement of panels (**a**,**b**) ensures maximum clarity and resolution for technical interpretation.

High-quality genomic DNA extraction facilitated successful allelic discrimination for all 65 participants. Genotyping results were rigorously validated via automated capillary electrophoresis, which provided clear differentiation between wild-type and variant alleles (Figure 1). Genotype frequencies were assessed for Hardy–Weinberg equilibrium (HWE); the distributions for *SLCO1B1* c.388A>G and our primary variant of interest, *SLCO1B3* c.699G>A, adhered to equilibrium (*p* > 0.05). Although *SLCO1B*1 c.521T>C and *SLCO1B3* c.334T>G exhibited deviations from HWE (*p* < 0.05), the use of a high-precision QIAxcel platform suggests that these deviations likely reflect the modest size and specific characteristics of this clinical cohort rather than technical inconsistencies.

Baseline demographics and clinical characteristics are detailed in Table 1. Patients who developed hyperbilirubinemia tended to be older (66.1 ± 13.4 vs. 58.7 ± 14.5 years), though this did not reach statistical significance (*p* = 0.081). Gender and ribavirin (RBV) co-administration were not identified as independent risk factors (*p* = 0.106 and *p* = 0.770, respectively). Notably, non-cirrhotic status was more prevalent among hyperbilirubinemic patients (91.7%) compared to the stable group (64.1%), demonstrating borderline significance (*p* = 0.086).

### 3.2. Temporal Dynamics and Clinical Resolution

Longitudinal analysis revealed a distinct temporal signature for drug-induced bilirubin elevation. In the vast majority of cases (91.7%; *n* = 11/12), hyperbilirubinemia was detected within the first four weeks of therapy. This early-onset pattern is consistent with immediate pharmacological interference with hepatic uptake mechanisms following the initiation of treatment.

While elevated bilirubin levels persisted until the End of Treatment (EOT, Week 12) in 45.4% (*n* = 5) of these patients, the biochemical shift was entirely reversible. By Week 24 (SVR12), 100% of the affected patients exhibited normalized bilirubin levels. This complete resolution confirms that the observed hyperbilirubinemia represents a transient, functional interference with sinusoidal transport mechanisms rather than sustained or structural hepatocellular injury.

### 3.3. Biochemical Markers and Mechanistic Insights

Comparative laboratory evaluations (Table 2) revealed a pivotal divergence in transaminase responses between the two groups. Patients who developed hyperbilirubinemia exhibited significantly higher AST levels (54.0 ± 47.3 vs. 25.1 ± 14.0 U/L; *p* < 0.001), whereas ALT levels remained stable and statistically comparable between the groups (*p* = 0.151).

This “AST-dominant” elevation, occurring in the absence of a corresponding ALT rise, provides strong mechanistic evidence that the bilirubin increase stems from pharmacological interference with transport mechanisms at the sinusoidal membrane rather than acute hepatocellular necrosis. This biochemical signature serves as a critical clinical differentiator, suggesting that OPrD-induced bilirubin spikes are functional and reversible rather than indicative of structural liver injury.

### 3.4. Pharmacogenomic Determinants: The Predominance of OATP1B3 c.699G>A

Genotypic and allelic distributions for all investigated loci are detailed in Table 3. Among the candidate variants, the *SLCO1B3* c.699G>A (rs7311358) polymorphism emerged as the sole significant genetic determinant for treatment-induced hyperbilirubinemia (*p* = 0.007). To identify the most parsimonious inheritance pattern, multiple genetic association models were rigorously evaluated using objective criteria.

To identify the most parsimonious inheritance pattern for the *OATP1B3* c.699G>A variant, multiple genetic association models were rigorously evaluated (Table 4). The comparison included Codominant, Dominant, and Recessive models. Our results demonstrated that the Dominant model (GG vs. GA + AA) provided the strongest statistical association and yielded the lowest Akaike Information Criterion (AIC: 61.4) and Bayesian Information Criterion (BIC: 63.5) values compared to the other models. These objective criteria confirm that the Dominant model is the best-fit model for explaining the genetic risk in our patient cohort.

Under this best-fit model, individuals harboring the c.699G>A variant allele exhibited a 6.3-fold increased risk of developing hyperbilirubinemia (OR: 6.30; 95% CI: 1.48–26.80; *p* = 0.032). These findings underscore the high specificity of the *OATP1B3* c.699G>A variant as a robust clinical biomarker for identifying patient susceptibility to OPrD-induced bilirubin spikes.

## 4. Discussion

Our results demonstrate that the *SLCO1B3* c.699G>A variant serves as a significant genetic predictor of hyperbilirubinemia in chronic hepatitis C (CHC) patients undergoing OPrD therapy. The data indicate that carriers of this polymorphism face a 6.3-fold increased risk of bilirubin elevation, likely stemming from a synergistic interaction between baseline transporter impairment and pharmacological inhibition by the paritaprevir component of the DAA regimen.

Hepatic bilirubin homeostasis depends on efficient sinusoidal uptake mediated by proteins encoded by *SLCO1B1* and *SLCO1B3* [1,5]. When these pathways are compromised by genetic variants or competitive drug–drug interactions, systemic bilirubin levels inevitably rise [10,11]. This mechanism is exemplified by Rotor syndrome, where the total absence of functional OATP1B1 and OATP1B3 leads to chronic conjugated hyperbilirubinemia [7]. Our findings extend this paradigm to antiviral treatment, suggesting that the c.699G>A variant may functionally reduce transporter capacity, thereby lowering the threshold for paritaprevir-induced inhibition [9,16].

The pivotal finding in our cohort was the “AST-dominant” biochemical signature, where bilirubin and AST levels rose independently of ALT. This specific discordance is a critical clinical differentiator; it suggests that the observed hyperbilirubinemia reflects competitive transport interference at the sinusoidal interface rather than acute hepatocellular necrosis [10,17]. The specific elevation of AST without a concomitant rise in ALT is a significant mechanistic insight. While ALT is predominantly a cytoplasmic enzyme highly specific to hepatocytes, AST is also localized within the mitochondria and can be transported across the sinusoidal membrane. The ‘AST-dominant’ profile observed here suggests that OATP1B3 might play a role in the equilibrium of specific non-bilirubin organic anions, including certain mitochondrial metabolites. Its pharmacological inhibition likely leads to a transient, non-lytic leakage or an equilibrium shift in mitochondrial enzymes rather than a loss of membrane integrity. This is consistent with the role of OATP1B3 in the hepatic uptake of a broad spectrum of endogenous compounds beyond bilirubin, including conjugated bile acids, where a blockade can disrupt mitochondrial enzyme kinetics and cellular anion homeostasis [18,19]. This discordance confirms that the hyperbilirubinemia is a result of physicochemical interference at the transporter level rather than hepatocellular necrosis, which would otherwise trigger a massive and parallel ALT release.

In drug-induced liver injury (DILI), one would typically expect a parallel and often more pronounced rise in ALT. The transient nature of these elevations in our study, manifesting within the first month and resolving completely by Week 24, further supports a non-necrotic etiology. In line with previous reports, such fluctuations appear clinically benign and seldom necessitate treatment cessation [9,20]. Furthermore, while ribavirin is known to induce hemolysis, its impact in our study was negligible, reinforcing the conclusion that OATP-mediated uptake interference, rather than increased bilirubin production, is the primary driver of bilirubin spikes during OPrD therapy.

In contrast to the strong association observed with *SLCO1B3* c.699G>A, *SLCO1B1* variants (c.388A>G and c.521T>C) showed no significant correlation with hyperbilirubinemia. This divergence from neonatal studies, where *SLCO1B1* is a major player in neonatal jaundice, highlights a potential developmental or stress-induced shift in transporter dependence [5,21,22,23]. While neonates rely heavily on OATP1B1 due to an immature conjugation system, our results suggest that in adults challenged by protease inhibitors, OATP1B3 assumes a more critical compensatory role in bilirubin clearance. Our identified allele frequencies align with established Caucasian population data, reinforcing the broader applicability of these findings [24,25].

Interestingly, non-cirrhotic patients exhibited hyperbilirubinemia more frequently (91.7%) than those with compensated cirrhosis. Although this trend was at the threshold of significance (*p* = 0.086), it prompts intriguing questions regarding hepatic remodeling. In advanced cirrhosis, baseline OATP expression is often already downregulated, as chronic hepatic remodeling compromises the transport system even before pharmacological challenge [26]. Therefore, the incremental inhibitory effect of paritaprevir might be statistically and clinically less discernible in a system where transport capacity is already chronically compromised. Alternatively, the altered bilirubin kinetics inherent to the cirrhotic liver may mask the functional impact of these genetic polymorphisms. This suggests that pharmacogenetic screening might be particularly valuable in non-cirrhotic patients where baseline transport is intact but susceptible to acute pharmacological blockade.

In our cohort, *SLCO1B1* c.521T>C and *SLCO1B3* c.334T>G exhibited deviations from Hardy–Weinberg Equilibrium (HWE). It is important to note that the genotype frequencies observed in this study are fully consistent with the baseline genetic characterization of this specific patient group, as previously reported and validated in our recent work [15]. The persistence of these frequencies across different analyses of the same cohort confirms that the HWE deviation is a stable, population-specific genetic profile of our study group rather than a result of genotyping artifacts. Furthermore, these frequencies remain in alignment with the gnomAD database [27], reinforcing that the observed distributions, while deviating from theoretical equilibrium, reflect the actual genetic landscape of the recruitment region.

### Limitations and Future Perspectives

Despite these significant findings, certain limitations of the present study must be acknowledged. The relatively small sample size may limit the generalizability of the results, although the achieved statistical power (0.81) confirms the robustness of the identified association for our primary variant. Additionally, the lack of OPrD plasma concentration measurements precluded a direct correlation between drug exposure levels and the observed genotypes. However, the implementation of high-resolution capillary electrophoresis (QIAxcel system) for genotyping ensures high technical reliability and eliminates the risk of visual interpretation bias inherent in traditional methods. While the modest sample size of this study is a limitation, the high statistical significance (*p* = 0.007) and the substantial Odds Ratio (6.30) observed for the *OATP1B3* c.699G>A variant underscore its potency as a robust genetic marker. Consequently, this research serves as a ‘Proof-of-Concept’ study, demonstrating that the functional impact of this polymorphism is sufficiently powerful to be detected even within a real-world clinical cohort. This suggests that the *OATP1B3* genotype could be a reliable predictor of drug-induced bilirubin shifts, warranting further validation in larger, multi-center prospective trials

Future investigations should prioritize expanded and diverse cohorts to substantiate our findings, potentially facilitating the integration of *OATP* variants into comprehensive polygenic risk scores for DAA therapy. From a clinical perspective, our findings serve as a “pharmacogenetic safety compass.” By identifying the *SLCO1B3* c.699G>A genotype prior to the initiation of therapy, clinicians can anticipate these “pseudo-toxic” bilirubin spikes. This proactive approach is vital to prevent clinical anxiety and unwarranted treatment discontinuations, a phenomenon often described as therapeutic inertia.

In the era of highly effective DAA regimens, ensuring treatment continuity is paramount. Distinguishing benign, genetically driven biochemical shifts from true drug-induced liver injury (DILI) is essential for achieving optimal sustained virological response (SVR) rates and ensuring patient safety in real-world clinical practice.

## 5. Conclusions

In conclusion, our study identifies the *SLCO1B3* c.699G>A variant as a robust genetic predictor of treatment-induced hyperbilirubinemia in patients receiving OPrD therapy. Through rigorous model evaluation, the dominant inheritance pattern was established as the best-fit model, demonstrating that carriers of the A-allele face a 6.3-fold increased risk of bilirubin elevation. The observed “AST-dominant” biochemical signature, characterized by isolated bilirubin and AST rises in the absence of significant ALT fluctuations, strongly underscores a transporter-mediated interference at the sinusoidal interface rather than direct hepatocellular injury or hepatotoxicity.

As the landscape of hepatology moves toward an era of personalized medicine, pharmacogenomic profiling of the *SLCO1B3* locus offers a valuable tool for tailoring antiviral strategies. Incorporating the assessment of OATP1B3 variants into clinical practice could empower clinicians to preemptively identify high-risk individuals, optimize monitoring protocols, and eliminate diagnostic uncertainty regarding “pseudo-toxic” biochemical shifts. While our findings provide strong clinical evidence and meet the requisite statistical power, further large-scale studies and functional analyses are warranted to fully elucidate the long-term impact of OATP-mediated transport on drug-induced biochemical dynamics and to refine personalized treatment algorithms across diverse global populations.

## Figures and Tables

**Table 1:** Baseline Demographic and Clinical Characteristics of Patients with Chronic Hepatitis C, Stratified by the Occurrence of Hyperbilirubinemia During OPrD Therapy.

Characteristics	Without Hyperbilirubinemia (*n* = 53)	With Hyperbilirubinemia (*n* = 12)	*p*-Value
Liver Status, *n* (%)			0.086
Non-cirrhotic	34 (64.1)	11 (91.7)	
Cirrhotic	19 (35.9)	1 (8.3)	
Gender, *n* (%)			0.106
Male	33 (62.3)	4 (33.3)	
Female	20 (37.7)	8 (66.7)	
Age (years), mean ± SD	58.7 ± 14.5	66.1 ± 13.4	0.081
Ribavirin Use, *n* (%)			0.770
With Ribavirin	8 (15.1)	3 (25.0)	
Without Ribavirin	45 (84.9)	9 (75.0)	

Notes: Data are expressed as *n* (%) or mean ± standard deviation (SD). *p*-values represent statistical comparisons between patients with and without hyperbilirubinemia, utilizing the Chi-square (χ^2^) test or Fisher’s exact test for categorical variables and Student’s *t*-test or Mann–Whitney U test for continuous variables. OPrD: ombitasvir/paritaprevir/ritonavir plus dasabuvir.

**Table 2:** Comparative Analysis of Biochemical and Metabolic Parameters Between Patients with and without Hyperbilirubinemia.

Variable	Without Hyperbilirubinemia (*n* = 53)	With Hyperbilirubnemia (*n* = 12)	*p*-Value
HCV-RNA (IU/mL)	2,581,116 ± 3,090,907	1,177,172 ± 1,617,990	0.133
**Liver Enzymes (U/L)**			
AST	25.1 ± 13.9	54.0 ± 47.3	**<0.001**
ALT	24.7 ± 17.4	33.2 ± 21.5	0.151
**Metabolic Parameters**			
Fasting Glucose (mg/dL)	106.6 ± 40.1	142.4 ± 65.6	**0.017**
Insulin (µU/mL)	14.6 ± 11.1	11.8 ± 10.9	0.455
HOMA-IR	3.9 ± 3.7	3.6 ± 2.9	0.794
**Lipid Profile (mg/dL)**			
Total Cholesterol	166.1 ± 34.2	154.4 ± 25.3	0.273
LDL-Cholesterol	92.3 ± 33.3	77.6 ± 23.0	0.155
HDL-Cholesterol	47.8 ± 15.6	58.1 ± 14.6	**0.042**
Triglycerides	139.4 ± 90.6	93.6 ± 35.5	0.092

Notes: Data are presented as mean ± standard deviation (SD). *p*-values denote statistical comparisons between groups using Student’s *t*-test or Mann–Whitney U test. Bold values indicate statistical significance (*p* < 0.05). HCV: hepatitis C virus; AST: aspartate aminotransferase; ALT: alanine aminotransferase; HOMA-IR: Homeostatic Model Assessment for Insulin Resistance; LDL: low-density lipoprotein; HDL: high-density lipoprotein.

**Table 3:** Association between OATP Genetic Polymorphisms and OPrD-Induced Hyperbilirubinemia.

Variant/Genetic Model	Genotype/ Allele	Normal Bilirubin (*n* = 53)	High Bilirubin (*n* = 12)	*p*-Value	OR (95% CI)	AIC	BIC
OATP1B1 c.388A>G				**0.210**			
Codominant	AA/AG/GG	26/19/8	3/5/4	0.244	Ref/2.2/4.3	68.4	72.8
Dominant	AA vs. AG + GG	26 vs. 27	3 vs. 9	0.200	2.89 (0.69–12.0)	67.2	69.4
Recessive	AA + AG vs. GG	45 vs. 8	8 vs. 4	0.207	2.81 (0.68–11.5)	68.1	70.3
Alleles	A/G	71/35	11/13	**0.080**	2.40 (0.91–6.29)	-	-
OATP1B1 c.521T>C				0.949			
Codominant	TT/TC/CC	41/7/5	9/2/1	0.941	Ref/1.3/0.9	67.5	71.8
Dominant	TT vs. TC + CC	41 vs. 12	9 vs. 3	0.905	1.14 (0.25–5.07)	66.2	68.4
Recessive	TT + TC vs. CC	48 vs. 5	11 vs. 1	0.901	0.87 (0.09–8.17)	67.4	69.5
Alleles	T/C	89/17	20/4	0.952	1.05 (0.32–3.43)	-	-
OATP1B3 c.334T>G				0.503			
Codominant	TT/TG/GG	7/18/28	2/2/8	0.491	Ref/0.3/1.1	67.9	72.3
Dominant	TT vs. TG + GG	7 vs. 46	2 vs. 10	0.677	1.31 (0.23–7.31)	67.2	69.4
Recessive	TT + TG vs. GG	25 vs. 28	4 vs. 8	0.502	1.78 (0.47–6.72)	66.8	69.0
Alleles	T/G	32/74	6/18	0.659	1.30 (0.47–3.56)	-	-
OATP1B3 c.699G>A				**0.007**			
Codominant	GG/GA/AA	44/9/0	7/3/2	0.021	Ref/2.1/8.4	64.2	67.8
Dominant *	GG vs. GA + AA	44 vs. 9	7 vs. 5	**0.009**	6.30 (1.48–26.8)	61.4	63.5
Recessive	GG + GA vs. AA	53 vs. 0	10 vs. 2	0.032	3.12 (0.7–14.1)	68.5	70.4
Alleles	G/A	97/9	17/7	**0.009**	4.44 (1.42–13.8)	-	-

Notes: Data are expressed as *n* (%). *p*-values represent statistical significance across the investigated models. OR: Odds Ratio; CI: Confidence Interval; AIC: Akaike Information Criterion; BIC: Bayesian Information Criterion. OPrD: ombitasvir/paritaprevir/ritonavir plus dasabuvir. *p*-values represent statistical significance across the investigated models. Bold values indicate statistical significance (*p* < 0.05). * Indicates the best-fit model identified by the lowest AIC and BIC values.

**Table 4:** Comparison of Genetic Association Models for the OATP1B3 c.699G>A Polymorphism.

Genetic Model	Genotypes	OR (95% CI)	*p*-Value
Codominant	GG vs. GA vs. AA	Ref/2.11/8.44	0.021
Dominant *	GG vs. GA + AA	6.30 (1.48–26.8)	0.009
Recessive	GG + GA vs. AA	3.12 (0.7–14.1)	0.032

Notes: OR: Odds Ratio; CI: Confidence Interval; AIC: Akaike Information Criterion; BIC: Bayesian Information Criterion. *p*-values denote the statistical significance of each genetic association model. * Indicates the best-fit model identified by the lowest AIC and BIC values. for the *OATP1B3* c.699G>A variant.

## Data Availability

The datasets generated and/or analyzed during the current study are available from the corresponding author upon reasonable request.

## Supplementary Materials

The following supporting information can be downloaded at: https://www.mdpi.com/article/10.3390/cimb48050452/s1.

## References

[1] Sticova E., Jirsa M. (2013). New insights in bilirubin metabolism and their clinical implications. World J. Gastroenterol..

[2] Wolkoff A.W., Samuelson A.C., Johansen K.L., Nakata R., Withers D.M., Sosiak A. (1987). Influence of Cl- on organic anion transport in short-term cultured rat hepatocytes and isolated perfused rat liver. J. Clin. Investig..

[3] Wolkoff A.W., Goresky C.A., Sellin J., Gatmaitan Z., Arias I.M. (1979). Role of ligandin in transfer of bilirubin from plasma into liver. Am. J. Physiol..

[4] Choudhuri S., Klaassen C.D. (2020). Elucidation of OATP1B1 and 1B3 transporter function using transgenic rodent models and commonly known single nucleotide polymorphisms. Toxicol. Appl. Pharmacol..

[5] Cui Y., König J., Leier I., Buchholz U., Keppler D. (2001). Hepatic uptake of bilirubin and its conjugates by the human organic anion transporter SLC21A6. J. Biol. Chem..

[6] Roy-Chowdhury N., Wang X., Roy-Chowdhury J., Pyeritz R., Korf B., Grody W. (2019). Bile pigment metabolism and its disorders. Emery and Rimoin’s Principles and Practice of Medical Genetics and Genomics: Cardiovascular, Respiratory, and Gastrointestinal Disorders.

[7] van de Steeg E., Stranecky V., Hartmannova H., Nosková L., Šebesta I., Jirásek T., Zeman J., Keppler D., Jirsa M., Schinkel A.H. (2012). Complete OATP1B1 and OATP1B3 deficiency causes human Rotor syndrome by interrupting conjugated bilirubin reuptake into the liver. J. Clin. Investig..

[8] Sticova E., Lodererova A., van de Steeg E., Frankova S., Jirsa M., Lanska V., Sperl J. (2015). Down-regulation of OATP1B proteins correlates with hyperbilirubinemia in advanced cholestasis. Int. J. Clin. Exp. Pathol..

[9] Cada D.J., Leonard J., Levien T.L., Baker D.E. (2015). Ombitasvir/Paritaprevir/Ritonavir and Dasabuvir. Hosp. Pharm..

[10] Campbell S.D., de Morais S.M., Xu J.J. (2004). Inhibition of human organic anion transporting polypeptide OATP 1B1 as a mechanism of drug-induced hyperbilirubinemia. Chem. Biol. Interact..

[11] Tátrai P., Krajcsi P. (2020). Prediction of Drug-Induced Hyperbilirubinemia by In Vitro Testing. Pharmaceutics.

[12] Nie Y., Yang J., Liu S., Wang X.J., Xu W., Zhang W. (2020). Genetic polymorphisms of human hepatic OATPs: Functional consequences and effect on drug pharmacokinetics. Xenobiotica.

[13] Degasperi E., Spinetti A., Lombardi A., Landonio S., Rossi L., Colombo S., Pasulo L., Aghemo A., Colombo M., Lampertico P. (2019). Real-life effectiveness and safety of sofosbuvir/velpatasvir/voxilaprevir in hepatitis C patients with previous DAA failure. J. Hepatol..

[14] Johnson A.D., Kavousi M., Smith A.V., Chen M.H., Dehghan A., Aspelund T., You L., Sigurdsson S., Launer L.J., Harris T.B. (2009). Genome-wide association meta-analysis for total serum bilirubin levels. Hum. Mol. Genet..

[15] Altintas Z., Altintas E. (2025). The Impact of Organic Anion-Transporting Polypeptide (OATP) Variants on the Side Effects of Direct-Acting Antivirals in Hepatitis C Patients. Cureus.

[16] Yang F., Liu L., Chen L., Jiang G.Z., Wang M.X., Du X.Y. (2018). OATP1B3 (699G>A) and CYP2C9*2, *3 significantly influenced the transport and metabolism of glibenclamide and glipizide. Sci. Rep..

[17] Chang J.H., Plise E., Cheong J., Ho Q., Lin M. (2013). Evaluating the in vitro inhibition of UGT1A1, OATP1B1, OATP1B3, MRP2, and BSEP in predicting drug-induced hyperbilirubinemia. Mol. Pharm..

[18] Kalliokoski A., Niemi M. (2009). Impact of OATP transporters on pharmacokinetics. Br. J. Pharmacol..

[19] Pan Q., Zhu G., Xu Z., Zhu J., Ouyang J., Tong Y., Chai J. (2023). Organic Anion Transporting Polypeptide (OATP) 1B3 is a Significant Transporter for Hepatic Uptake of Conjugated Bile Acids in Humans. Cell Mol. Gastroenterol. Hepatol..

[20] Deeks E.D. (2015). Ombitasvir/Paritaprevir/Ritonavir Plus Dasabuvir: A Review in Chronic HCV Genotype 1 Infection. Drugs.

[21] Huang M.J., Kua K.E., Teng H.C., Tang K.S., Weng H.W., Huang C.S. (2004). Risk factors for severe hyperbilirubinemia in neonates. Pediatr. Res..

[22] Liu J., Long J., Zhang S., Fang X., Luo Y. (2013). The impact of SLCO1B1 genetic polymorphisms on neonatal hyperbilirubinemia: A systematic review with meta-analysis. J. Pediatr..

[23] Atasilp C., Kanjanapipak J., Vichayaprasertkul J., Jinda S., Khemawoot P., Rochanawutanon M., Tanomsri P., Sukasem C. (2022). Associations between UGT1A1 and SLCO1B1 polymorphisms and susceptibility to neonatal hyperbilirubinemia in Thai population. BMC Pediatr..

[24] Smith N.F., Marsh S., Scott-Horton T.J., Hamada A., Mross K., McLeod H.L., Figg W.D. (2007). Variants in the SLCO1B3 gene: Interethnic distribution and association with paclitaxel pharmacokinetics. Clin. Pharmacol. Ther..

[25] Sanna S., Busonero F., Maschio A., McArdle P.F., Usala G., Dei M., Lai S., Mulas A., Piras M.G., Curreli N. (2009). Common variants in the SLCO1B3 locus are associated with bilirubin levels and unconjugated hyperbilirubinemia. Hum. Mol. Genet..

[26] Thakkar N., Slizgi J.R., Brouwer K.L.R. (2017). Effect of Liver Disease on Hepatic Transporter Expression and Function. J. Pharm. Sci..

[27] Karczewski K.J., Francioli L.C., Tiao G., Cummings B.B., Alföldi J., Wang Q., MacArthur D.G. (2020). The mutational constraint spectrum quantified from variation in 141,456 humans. Nature.

