# Supplementary material for: OATP1B3 c.699G>A Predicts a 6.3-Fold Increased Risk of Hyperbilirubinemia During OPrD Therapy for HCV
Source: Curr Issues Mol Biol. 2026 Apr 27;48(5):452. doi: 10.3390/cimb48050452 (PMC13204828; doi:10.3390/cimb48050452)
Supplement: Supplementary file 1 [file cimb-48-00452-s001.zip › cimb-4251433-supplementary.pdf]

Supplementary Table S1: Primer pairs and PCR-RFLP evaluation method used for the amplification of *SLOC1B1* and *SLCO1B3*.

Variation		Primer	Temperature of annealing	Restriction endonuclease	PCR products
SLOC1B1	c.388 A>G variation (rs2306283)	Primer F 5′-CTGTGTTGTTAATGGGCGAA-3′	57.0°C	TaqI	A allele: 159, 247 bp; G allele: 23, 136, 247 bp
		Primer R 5′-GGGGAAGATAATGGTGCAAA-3′			
	c.521T>C variation (rs4149056)	Primer F 5′-TTGTCAAAGTTTGCAAAGTG-3′ C	52.0°C	Hin6I	T allele: 209 bp; C allele: 21,188 bp
		Primer R 5′-GAAGCATATCCATGAGC -3′			
SLOC1B3	c.334T>G variation (rs4149117)	Primer F 5′-GAAGGTACAATGTCTTGGGC-3′	51.0°C	AluI	T allele: 86, 253 bp; G allele: 126, 213 bp
		Primer R 5′-CTCTCAAAAGGTAACTGCCC-3′			
	699 G>A variation (rs7311358)	Primer F 5′-ATGATTACATTCCCTGGATC-3′	55.0°C	RsaI	G allele: 61, 242 bp; A allele: 28, 275 bp
		Primer R 5′-ACTATCATGGTACCTTGTTC-3′			

Notes: F, forward; R, reverse; bp, base pairs; *SLCO1B1*, solute carrier organic anion transporter family member 1B1; *SLCO1B3*, solute carrier organic anion transporter family member 1B3. All primer sequences are presented in the 5′–3′ orientation. Annealing temperatures (°C) and restriction endonucleases were optimized for the RFLP-based genotyping of each variant.
